# Joint statement on evidence-based practices in mechanical ventilation: suggestions from two Brazilian medical societies

**DOI:** 10.62675/2965-2774.20250242-en

**Published:** 2025-01-22

**Authors:** Juliana Carvalho Ferreira, Arthur Oswaldo de Abreu Vianna, Bruno Valle Pinheiro, Israel Silva Maia, Sérgio Vasconcellos Baldisserotto, Alexandre Marini Isola

**Affiliations:** 1 Hospital das Clínicas Faculdade de Medicina Universidade de São Paulo São Paulo SP Brazil Division of Pulmonology, Instituto do Coração, Hospital das Clínicas, Faculdade de Medicina, Universidade de São Paulo - São Paulo (SP), Brazil.; 2 Rede D’Or São Luiz Rio de Janeiro RJ Brazil Clínica São Vicente, Rede D’Or São Luiz - Rio de Janeiro (RJ), Brazil.; 3 Department of Internal Medicine Faculdade de Medicina Universidade Federal de Juiz de Fora Juiz de Fora MG Brazil Hospital Universitário, Department of Internal Medicine, Faculdade de Medicina, Universidade Federal de Juiz de Fora - Juiz de Fora (MG),Brazil.; 4 Department of Internal Medicine Universidade Federal de Santa Catarina Florianópolis SC Brazil Department of Internal Medicine, Universidade Federal de Santa Catarina - Florianópolis (SC),Brazil.; 5 Hospital Nereu Ramos Florianópolis SC Brazil Hospital Nereu Ramos - Florianópolis (SC), Brazil.; 6 Santa Casa de Misericórdia de Porto Alegre Porto Alegre RS Brazil Santa Casa de Misericórdia de Porto Alegre - Porto Alegre (RS), Brazil.; 7 Department of Continuing Education Imed Group São Paulo SP Brazil Department of Continuing Education, Imed Group - São Paulo (SP), Brazil

**Keywords:** Respiration, artificial, Practice guidelines as topic, Noninvasive ventilation, Ventilator weaning, Intensive care units

## Abstract

Mechanical ventilation can be a life-saving intervention, but its implementation requires a multidisciplinary approach, with an understanding of its indications and contraindications due to the potential for complications. The management of mechanical ventilation should be part of the curricula during clinical training; however, trainees and practicing professionals frequently report low confidence in managing mechanical ventilation, often seeking additional sources of knowledge. Review articles, consensus statements and clinical practice guidelines have become important sources of guidance in mechanical ventilation, and although clinical practice guidelines offer rigorously developed recommendations, they take a long time to develop and can address only a limited number of clinical questions. The *Associação de Medicina Intensiva Brasileira* and the *Sociedade Brasileira de Pneumologia e Tisiologia* sponsored the development of a joint statement addressing all aspects of mechanical ventilation, which was divided into 38 topics. Seventy-five experts from all regions of Brazil worked in pairs to perform scoping reviews, searching for publications on their specific topic of mechanical ventilation in the last 20 years in the highest impact factor journals in the areas of intensive care, pulmonology, and anesthesiology. Each pair produced suggestions and considerations on their topics, which were presented to the entire group in a plenary session for modification when necessary and approval. The result was a comprehensive document encompassing all aspects of mechanical ventilation to provide guidance at the bedside. In this article, we report the methodology used to produce the document and highlight the most important suggestions and considerations of the document, which has been made available to the public in Portuguese.

## INTRODUCTION

Invasive and noninvasive mechanical ventilation (MV) is essential in the treatment of patients with acute respiratory failure and is the most frequently implemented support measure in intensive care units (ICUs).^(1,2)^ Although it is a life-saving measure, MV requires an understanding of its indications, contraindications, and management, as it can be associated with complications, especially when it is implemented inappropriately.^(3)^ Because it is used mainly in severe or potentially severe patients, it involves complex coordination between healthcare providers, including respiratory therapists, nurses, physicians, and other specialists, to ensure optimal patient care, proper ventilator management and timely interventions to avoid complications.

The management of MV is a core competency in critical care training and should be part of the undergraduate curricula in medicine, nursing and physiotherapy, as well as residency and subspecialization in critical care.^(4)^ However, trainees and practicing professionals often report low confidence in managing MV patients and performing basic adjustments^(5,6)^and seek other sources of knowledge about MV. Since the 1990s, review articles and consensus statements on MV have become important sources of guidance for clinicians.^(7)^ In recent years, most consensuses have employed the Grading of Recommendations Assessment, Development and Evaluation (GRADE) methodology^(8)^ to establish clinical practice guidelines.^(9-11)^ This methodology is accepted as the best strategy for providing recommendations based on evidence, but because extensive work is needed to formulate recommendations based on a limited number of clinical questions, it may not be suitable if the intention is to provide a comprehensive document encompassing all aspects of a broad topic, such as MV.

In 2013, the *Associação de Medicina Intensiva Brasileira* (AMIB) and the *Sociedade Brasileira de Pneumologia e Tisiologia* (SBPT) published the Brazilian Recommendations for Mechanical Ventilation.^(12,13)^ Twenty-nine topics related to MV and suggestions for MV management were given for most clinical situations. Although a systematic methodology such as the GRADE was not adopted, the document became an important source of guidance for clinicians in Brazil. It was published as a research article in two parts and as a manual in PDF, which could be consulted at the bedside. Since then, new studies have been conducted and published, as well as guidelines on different aspects of ventilatory support, coordinated by different medical societies.^(9-11,14-16)^ In addition, during the coronavirus disease 2019 (COVID-19) pandemic, when many patients required MV, the complexity of the conditions that require ventilatory support and the need for capacity building among healthcare professionals became clear.^(17)^

As a result, in 2023, AMIB and SBPT sponsored a project to update the recommendations. In this article, we report the methodology used to produce the document and highlight the most important suggestions and considerations of the document, which has been made available to the public (https://indd.adobe.com/view/017f739a-847f-4587-9bef-15b9c01756ba).

## METHODOLOGY

The Organizing Committee selected 38 topics related to MV for patients with respiratory failure and other indications of MV that were addressed in this document.

Each society indicated members who were considered experts in the field and involved in research and/or teaching of MV in Brazil to be invited to participate in the project. After a formal invitation and confirmation of those who were able to participate in the project, the group of experts was confirmed with 75 participants. The experts were all health care professionals specializing in intensive care, including physicians, nurses, physiotherapists, speech therapists, dentists, and nutritionists. They predominantly worked in the Southeast Region of Brazil (67%), with another 17% from the South Region, 10% from the Northeast Region, 5% from the Central West Region and 1% from the North Region. The participants were divided to work in pairs, and each topic was assigned to a pair of experts. The content to be addressed by each pair in their respective theme was previously determined by the organizing committee at the time of the invitation. Expertise and previous experience with their theme were taken into account when inviting each pair.

The pairs searched PubMed and the Cochrane Central Register of Controlled Trials databases for articles published on the topic. The search was limited to the last twenty years and focused on, but was not limited to, journals in the following areas: Intensive Care, Pulmonology, and Anesthesiology, including the journals of the respective Brazilian societies in these specialties: Critical Care Science (formerly *the Revista Brasileira de Medicina Intensiva*), the *Jornal Brasileiro de Pneumologia*, and the *Revista Brasileira de Anestesiologia*. Based on the results, each pair produced a text relevant to their topic and sent it to the organizing committee, with their respective bibliographic references. The format adopted to provide guidance was as follows: Comment (brief explanation of the topic to be addressed), followed by suggestions and considerations, as defined in table 1.

**Table 1:** Definitions of suggestions and considerations used in the document

Terms used	Definition and level of evidence	Example
Comment	Brief explanation of the topic to be addressed	The use of the prone position for patients under mechanical ventilation has gained prominence in the last decade due to the improvement in the clinical outcome of patients with severe and moderate ARDS
Suggestion	When the use of an intervention or monitoring is indicated, or not indicated, based on at least one randomized trial with low risk of bias or on at least one meta-analysis with low risk of bias	*“We suggest using a tidal volume of 4 - 8mL/kg of predicted weight for patients with ARDS”*
	Statements based on documents developed by national and international health authorities, such as the World Health Organization or Ministry of Health, and for statements based on well-established medical society guidelines, such as the Advanced Cardiovascular Life Support guidelines	“*Place all components to be sent for high-level disinfection (respiratory valve, active humidifier, flow sensor, and expiratory tube, if used, and other connectors and components) in a closed container designated for transport to the sterilization unit”*
Consideration	When the use of an intervention or monitoring is or is not to be considered, based on randomized studies or meta-analyses with high or undetermined risk of bias, observational studies (cohorts or case–controls) or the opinions of the experts	*“Consider the use of controlled initial respiratory rate between 12 - 16bpm, in the initial adjustment of the mechanical ventilator”*

ARDS - acute respiratory distress syndrome; bpm - breaths per minute.

In addition, we used “Suggestion” for statements based on documents developed by national and international health authorities, such as the World Health Organization or Ministry of Health, and for statements based on well-established medical society guidelines, such as the Advanced Cardiovascular Life Support (ACLS) guidelines.

The content prepared by each pair was then compiled and summarized by the Organizing Committee, which prepared all the topics for the pairs to present at a face-to-face meeting held on November 20 and 21, 2023, in Florianópolis, Santa Catarina, Brazil, prior to the Brazilian Congress of Intensive Care Medicine. During the meeting, all pairs presented their suggestions and considerations, submitting them to the evaluation and appreciation of all those present. The plenary held its manifestations freely, and all the suggestions were discussed. When there was no consensus and two alternatives for formulating suggestions/considerations remained after ample discussion, the two alternatives were presented for electronic voting using an anonymous system.

At the end of this stage, the organizing committee compiled the text sent by all the pairs and made the agreed-upon adjustments after the plenary session. The revised document was sent to each expert for review or final adjustments. Finally, the organizing committee reviewed the final edition of the unified document with all the themes.

The document included multidisciplinary topics, such as nursing, physiotherapy, nutrition, speech therapy, and dentistry. New topics were added, such as ventilation-induced lung injury (VILI), extracorporeal membrane oxygenation (ECMO), MV in pregnant women, MV in the transport of patients, ICU-acquired weakness, MV in palliative care patients and a specific topic for prone positioning. Table 2 shows the list of topics covered in the document and the most relevant suggestions and considerations for each topic.

**Table 2:** Highlights on each topic of the practices in mechanical ventilation from the Associação de Medicina Intensiva Brasileira and the Sociedade Brasileira de Pneumologia e Tisiologia

	Topic	Highlights
1	Indication of noninvasive and invasive ventilatory support*	**Regarding NIV in ARDS** **Consider:** − NIV can be performed in mild to moderate ARDS in selected locations with rigorous clinical monitoring of the response to avoid delays in intubation in case of failure − Do not use NIV in severe ARDS cases with PaO2/FiO2 < 100mmHg
2	Noninvasive strategies in acute respiratory failure	**Regarding the use of HFNC** **Suggested:** − Can be used as the first choice of respiratory support for mild to moderate hypoxemia with hemodynamic stability − Use postextubation, alone or in association with NIV for high-risk patients
3	Intubation and tracheostomy	**Regarding the evaluation of the patient to be intubated** **Suggested:** − Use a validated tool for the evaluation and identification of potential difficult airway in planning orotracheal intubation − Identify patients with anatomical and/or physiological difficult airways **Consider:** − Use videolaryngoscopy for patients with a MACOCHA airway score ≥ 3
4	Conventional ventilatory modes and initial adjustment of the invasive ventilator	**Regarding initial parameters and conduct of invasive MV** **Suggested** − Use predicted body weight for calculating prescribed VT − Use an initial tidal volume of 6 to 8mL/Kg of predicted body weight − Adjust PEEP and FiO2 relationships individually aiming for an SpO2 of 92 to 96%
5	Advanced ventilatory modes	**Regarding the indication of advanced modes** **Consider** − May use advanced ventilatory modes in individualized clinical situations provided the user is familiar with their adjustments and the patient’s clinical condition is thought to potentially benefit from the specific features of each mode
6	Patient–ventilator asynchrony	**Regarding asynchrony diagnosis** **Consider:** − Search for the presence of asynchronies and their corrections during the evaluation of the patient on MV, observing the frequency of occurrence and types.
7	Monitoring the patients under ventilatory support†	**Regarding ventilatory mechanics monitoring** **Suggested:** − Monitor the presence and value of auto-PEEP regularly **Consider:** − Regularly monitor the respiratory system mechanics, especially in conditions like ARDS, as maintaining parameters at safe levels is associated with lower mortality − Parameters to monitor: Ppeak, Pplat, Pres, DP, auto-PEEP, Rwa, and CSR
8	Monitoring gas exchange	**Regarding arterial blood gases:** **Consider:** − Collect arterial blood gases for all patients on MV approximately 20 minutes after adjusting ventilator parameters and daily during the acute phase.
9	Ventilator alarms	**Consider:** − Develop and ensure adherence to institutional protocols defining minimum adequate alarm adjustment parameters for all patients − From a predefined protocol, individualize alarm limits according to each patient and clinical condition to avoid alarm fatigue
10	Sedation, analgesia and neuromuscular blockade during MV	**Regarding sedation monitoring** **Consider** − For patients on neuromuscular blocking drugs, monitoring with simplified electroencephalogram equipment may be indicated, as scoring systems cannot determine the level of pain, sedation depth, or the presence of *delirium*
11	MV for asthma	**Regarding mechanical ventilation in asthma** **Consider:** − Use VCV or PCV modes − VT: 6– to 8mL/kg of predicted weight initially. Depending on the ventilatory mechanics, it may need to be reduced
12	MV for patients with COPD	**Regarding MV goals in acute exacerbation-COPD** **Consider** − During intubation, use the largest diameter cannula possible to reduce airway resistance and facilitate secretion removal − Adjust MV to improve oxygenation and ventilation, reduce ventilatory workload, and avoid dynamic hyperinflation − Systematic monitoring of ventilatory mechanics during exacerbation and, when necessary, during assisted ventilation and weaning − Active search for asynchronies, ineffective trigger, and auto-PEEP
13	Ventilator-associated pneumonia (VAP)	**Regarding preventive measures for VAP** **Consider:** − Using an orotracheal tube with subglottic aspiration for patients requiring more than 48 - 72 hours of MV − Monitoring endotracheal tube cuff pressure, maintaining values between 20 and 30cmH2O, especially during procedures like oral hygiene, position change, and prone position. There is no benefit in continuous or regular monitoring
14	MV for patients with ARDS	**Suggested:** − Adjust tidal volume to 4 - 8mL/kg (predicted weight), initially starting with 6mL/kg and adjusting according to plateau pressure, PaCO2, and pH − Use the lowest possible FiO2 to maintain SpO2 between 92% and 96% across all ARDS severity categories. − Adjust ventilatory parameters to limit plateau pressure to ≤ 30cmH2O. − Avoid using PEEP < 5 cmH2O for ARDS patients. − Avoid prolonged recruitment maneuvers for ARDS patients. **Consider:** − Use the 2023 definition for diagnosing and classifying ARDS severity. − Limit the driving pressure to less than or equal to 15cmH2O for all categories of ARDS severity
15	Ventilation in the prone position for intubated patients	**Suggested:** − Prone patients with PaO2/FiO2 ≤ 150mmHg with FiO2 > 60% and PEEP ≥ 5cmH2O as early as possible, preferably within the first 12 hours after stabilization and hypoxemia confirmation − Discontinue proning sessions when gas exchange improves (PaO2/FiO2 > 150mmHg for > 4 hours in supine position) or if two consecutive proning sessions decrease PaO2/FiO2 by > 20% compared to the supine position − Interrupt proning if complications arise
16	Preventing VILI	**Regarding VILI** **Suggested:** − Use intermediate tidal volumes (6 - 8mL/kg of predicted weight) for patients without ARDS at risk of developing ARDS − Use moderate PEEP levels (5 - 8cmH2O) for patients with normal lungs **Consider:** − Monitor patient muscle effort through inspiratory or expiratory pauses − Do not use mechanical power to guide the ventilatory strategy for patients in clinical practice − Jointly assess driving pressure and respiratory rate at the bedside
17	Extracorporeal circulation	**Suggested:** − Indicate ECMO for patients with hypoxemic acute respiratory failure with refractory hypoxemia despite optimized protective MV with PaO2/FiO2 < 50mmHg for > 3 hours OR PaO2/FiO2 < 80mmHg for > 6 hours − Use ECMO for patients with hypercapnic respiratory failure and pH < 7.25 associated with PaCO2 ≥ 60mmHg for > 6 hours despite optimization of protective ventilatory parameters − Do not routinely use nitric oxide for patients with acute respiratory failure and ARDS
18	MV for patients with thoracic trauma	**Consider:** − For more severe patients, particularly with ARDS or other severe clinical situations, initiate deep sedation and adequate analgesia, and start MV with assist-control modes (VCV or PCV) − PCV mode may be superior to VCV mode due to tighter control of maximum airway pressures
19	MV during surgical procedures	**Regarding preoperative evaluation** **Suggested:** − Use MV with VT of 8mL/kg of predicted weight (6 - 10mL/kg) for patients without acute lung injury. − For ARDS patients undergoing surgical procedures, use protective ventilation with VT of 6mL/kg of predicted weight **Consider:** − Assess all patients for the risk of postoperative pulmonary complications using a specific scale. The ASA classification is a subjective scale with low precision
20	MV for obese patients	**Suggested:** − Preventive use of NIV after extubation in obese patients **Consider:** − Objectively assess factors associated with difficult intubation − VT: 6 - 8mL/kg of predicted weight
21	MV for neurological patients	**Regarding PaO2 and PaCO2 targets** **Suggested:** − Ventilate all potential donors with protective lung ventilation **Consider:** − The ideal PaO2 target for patients with acute brain injury with or without intracranial hypertension should be between 80 and 120mmHg − The PaCO2 target for patients with acute brain injury with or without intracranial hypertension should be between 35 and 45mmHg
22	MV for neuromuscular patients	**Consider:** − NIV can be employed in acute respiratory failure, respecting contraindications and monitoring failure criteria − Avoid NIV for patients with neuromuscular disease and bulbar involvement or those with bronchial hypersecretion − Initiate MV in assist-control mode with tidal volume of 10mL/kg of predicted weight. Lower VTs are associated with atelectasis in the early days of MV. Subsequently, follow protective ventilation strategies with VT between 6 and 8mL/kg of predicted weight
23	MV for patients with heart disease	**Regarding NIV** **Suggested:** − Use NIV with CPAP or BiPAP for patients with signs of acute respiratory failure caused by cardiogenic pulmonary edema − Do not routinely use NIV for patients with cardiogenic shock − Employ NIV immediately after extubation (prophylactic NIV) to reduce the risk of extubation failure
24	MV for patients undergoing CPR	**Suggested:** − In nonintubated patients, maintain synchronous chest compression with ventilation at a 30:2 ratio. For patients with definitive airways, maintain asynchronous chest compression with ventilation, with 100 to 120 chest compressions/min and 8 to 10 ventilations/minute − Avoid hypoxia or hyperoxia during CPR, as it can worsen the prognosis of cardiac arrest victims − Monitor ETCO2 whenever possible with a target of 20mmHg
25	Weaning the patient from invasive MV	**Suggested:** − Daily evaluation of weaning readiness in all patients on MV for > 24 hours, i.e., readiness for an SBT − Use of a sedation protocol, which can be its daily interruption or adjustment according to established targets − Perform the SBT in PSV mode with a PS level between 5 and 7cmH2O with PEEP between 0 and 5cmH2O for 30– to 60 minutes − Apply preventive NIV immediately after extubation for patients at high risk of extubation failure − Apply facilitating NIV for patients with hypercapnic respiratory failure, particularly COPD exacerbation or neuromuscular disease, who fail the SBT − Do not use rescue NIV to avoid reintubation for patients developing acute respiratory failure after extubation
26	Patients with prolonged weaning‡ §	**Suggested:** − For prolonged weaning, perform the SBT in PSV mode with a PSV level between 5 and 7cmH2O with PEEP between 0 and 5cmH2O for 30– to 60 minutes **Consider:** − Define prolonged weaning as weaning not completed within 7 days after the first attempt to separate the patient from the ventilator − Before extubating patients who succeed in the SBT, return them to pretest ventilator parameters for approximately 1 hour to rest the patient and reduce the risk of exhaustion after extubation
27	Hemodynamic monitoring and treatment for patients under MV	**Suggested:** − The diagnosis of right and/or left ventricular dysfunction should be performed by echocardiogram to demonstrate the impact of respiratory pressures on the right chambers and the presence of left ventricular dysfunction contributing to pulmonary edema − Do not routinely use a pulmonary artery catheter in ARDS cases − As an adjunctive treatment for right ventricle dysfunction and refractory hypoxia, use the prone position. Use pulmonary vasodilators in selected cases
28	Speech therapy care in the rehabilitation of patients after MV	**Regarding specific care for patients postextubation** **Suggested:** − Implement a multidisciplinary approach for better identification, diagnosis, and treatment of dysphagia, ensuring greater safety in clinical management **Consider:** − Perform speech and swallowing evaluations for all patients who underwent prolonged intubation for ≥ 48 hours or reintubation and have clinical criteria postextubation within 24 - 48 hours
29	Nursing care for patients on invasive and noninvasive ventilatory support	**Suggested:** − Replace heat and moisture exchangers every 7 days (hygroscopic and hydrophobic), provided the device is maintained at the correct height and position relative to the endotracheal tube. In case of dirt, condensation, or damage, the filter should be replaced immediately − Do not routinely change the ventilator circuit; change it only when visible dirt, damage, or prolonged ventilation (> 30 days) occurs **Consider:** − Closed suction catheters should be used to prevent infections and avoid lung derecruitment
30	Physiotherapy care for patients on ventilatory support	**Suggested:** − Secretion removal therapies such as positioning, manual hyperinflation or ventilator, chest wall compression and oscillation should be used to improve oxygenation and secretion elimination in mechanically ventilated patients − Prior to physiotherapeutic care, a physiotherapeutic diagnosis should be made using tools for assessing peripheral muscle strength − Implement inspiratory muscle training for patients ventilated in the ICU for more than 7 days and for those who failed to wean from MV due to respiratory muscle weakness − Avoid the routine use of normal saline (isotonic) instillation during tracheal suctioning procedures, as it has shown potential adverse effects on oxygen saturation and cardiovascular stability, in addition to contributing to VAP **Consider:** − The appropriate dose of early mobilization is defined by clinical efficacy and individual tolerance
31	Nutritional care for patients under MV	**Suggestion:** − When available, it is suggested that the caloric needs of critically ill patients on MV be estimated by indirect calorimetry, considering the clinical condition and frequency of measurement. **Consider:** − Use protocols to guide nutritional therapy for critically ill patients on MV to improve nutritional adequacy outcomes and gastrointestinal symptom management − Perform nutritional screening for critically ill patients on MV within 24– to 48 hours of ICU admission. After identifying nutritional risk, perform a complete nutritional assessment. Use validated tools − Use the enteral route as the first option when the patient is adequately perfused and with a viable gastrointestinal tract
32	Weakness acquired in the ICU	**Suggested:** − There is no gold standard method for diagnosing ICU-acquired muscle weakness − Active early mobilization should be implemented to prevent ICU-acquired muscle weakness − Perform muscle rehabilitation for patients who have already reversed acute illness, have no electrolyte disturbances, and are being nutritionally supported within individual caloric-protein goals
33	Dental care for patients under MV	**Consider:** − Clean teeth with a small-headed soft brush − Clean alveolar ridges, cheek mucosa, lips, palate, tongue dorsum, and portion of the tracheal tube within the mouth with swabs or gauze − Create and implement care and therapeutic intervention protocols
34	Palliative respiratory support	**Regarding noninvasive support strategies** **Consider:** − Use supplemental oxygen for patients with dyspnea and associated hypoxemia and assess symptomatic response − Use NIV for patients with acute respiratory failure due to potentially reversible causes as a palliative technique to relieve dyspnea when the patient is not a candidate for intubation but wishes for other artificial procedures to prolong life − Perform a trial of NIV for terminal patients to provide additional time for patients to complete important end-of-life activities and say goodbye to family members **Regarding palliative extubation** **Suggested:** − Before palliative extubation, hold a family meeting to share information about the procedure, prepare for physical signs of discomfort that may occur, and how these will be monitored and treated, with a variable time until death **Consider:** − Use adjuvant medications preemptively to avoid uncomfortable symptoms associated with palliative extubation − Use a palliative extubation protocol
35	Transport of patients on MV	**Consider:** − A checklist related to necessary items for safe transport can be a guiding tool for the whole team. The patient profile, transport type, distance, and duration will determine the necessary materials, equipment, and team − Minimum required monitoring for safe transport includes: monitor with continuous electrocardiogram, heart rate, respiratory rate, blood pressure, oxygen saturation, ETCO2, and temperature
36	Mechanical ventilation during ICU procedures	**Regarding bronchoscopy** **Consider:** − Do not perform bronchoscopy on patients with hypoxemia that cannot be corrected with supplemental O2 − Use NIV for nonintubated and high-risk hypoxemic patients **Regarding upper digestive endoscopy** **Suggested:** − Monitor with pulse oximetry, blood pressure, and cardiac monitoring, and observe respiratory activity, consciousness level, and signs of discomfort at the bedside **Consider:** − Use HFNC to reduce hypoxemia risk
37	MV for pregnant women	**Regarding intubation** **Suggested:** − Safe ventilation strategies should be observed for pregnant women as for nonpregnant patients, respecting VT limits of 6 to 8mL/kg of predicted weight. In ARDS cases, use 4 to 8mL/kg of predicted weight **Consider:** − To avoid fetal hypoxemia, maintain maternal PaO2 ≥ 70mmHg or SatO2 ≥ 95%. Maintain PaCO2 > 30mmHg to avoid placental vasoconstriction − Assess the airway of the pregnant patient, observing the usual predictors of difficult airway. In this case, prefer intubation with videolaryngoscope assistance − During intubation, position the patient with an elevated backrest between 20 and 30º to prevent bronchoaspiration
38	Ventilatory support for patients with COVID-19	**Regarding oxygen therapy, NIV, and HFNC** **Suggested:** − Use VT of 4 - 8mL/kg of predicted weight − Do not perform routine recruitment maneuvers on patients with COVID-19-associated respiratory failure − Use prone position early in intubated patients with COVID-19 with PaO2/FiO2 < 150 with PEEP ≥ 5cmH2O for 16 - 20 hours **Consider:** − Measure DP and keep it ≤ 15cmH2O − Start respiratory support with oxygen use to maintain saturation between 92% and 96% − NIV, especially CPAP, has been shown to be superior to high-flow nasal cannula in avoiding orotracheal intubation in COVID-19 patients

Table 2 shows the most relevant suggestions and considerations for each topic. To access all the suggestions and considerations, please refer to the original document, which is freely available on the websites of the two societies: https://indd.adobe.com/view/017f739a-847f-4587-9bef-15b9c01756ba. *For topic 1, a vote was necessary to decide if a consideration to intubate patients with a Glasgow coma scale score of 8 or less was to be included (the decision was 65% in favor of using this cutoff); † for topic 7, a vote was necessary to decide if a table with suggested cutoff values was included in the topic (the decision was 73% in favor of including the table); ‡ for topic 26, a vote was necessary to decide between using the term liberation or weaning (the vote was in 80% favor of “weaning”); § for topic 26, a vote was necessary to decide between suggesting the use of PSV and considering the use of a T tube *versus* suggesting that either technique could be used (the vote was 58% in favor of suggesting PSV preferably).

NIV - noninvasive ventilation; ARDS - acute respiratory distress syndrome; PaO_2_/FiO_2_ - ratio of partial pressure of oxygen to the fraction of inspired oxygen; HFNC - high-flow nasal cannula; MV - mechanical ventilation; VT tidal volume PEEP - positive end-expiratory pressure; FiO_2_ - fraction of inspired oxygen; SpO_2_ - oxygen saturation; Ppeak - peak pressure; Pplat - plateau pressure; Pres - resistive pressure; DP - driving pressure; Raw – airway resistance; Crs - static compliance of the respiratory system; VCV - volume cycled ventilation; PCV - pressure-controlled ventilation; COPD - chronic obstructive pulmonary disease; VAP - ventilator-associated pneumonia; PaO2 - partial pressure of oxygen; PaCO_2_ - partial pressure of carbon dioxide; VILI - ventilator-induced lung injury; ECMO - extracorporeal membrane oxygenation; NO - nitric oxide; ASA - American Society of Anesthesiologists; CPAP - continuous positive airway pressure; BiPAP - bilevel positive airway pressure; CPR - cardiopulmonary resuscitation; ETCO_2_ - end-tidal carbon dioxide; SBT - spontaneous breathing trial; PSV - pressure support ventilation; ICU - intensive care unit.

### COMMENTS

**Figure 1:**
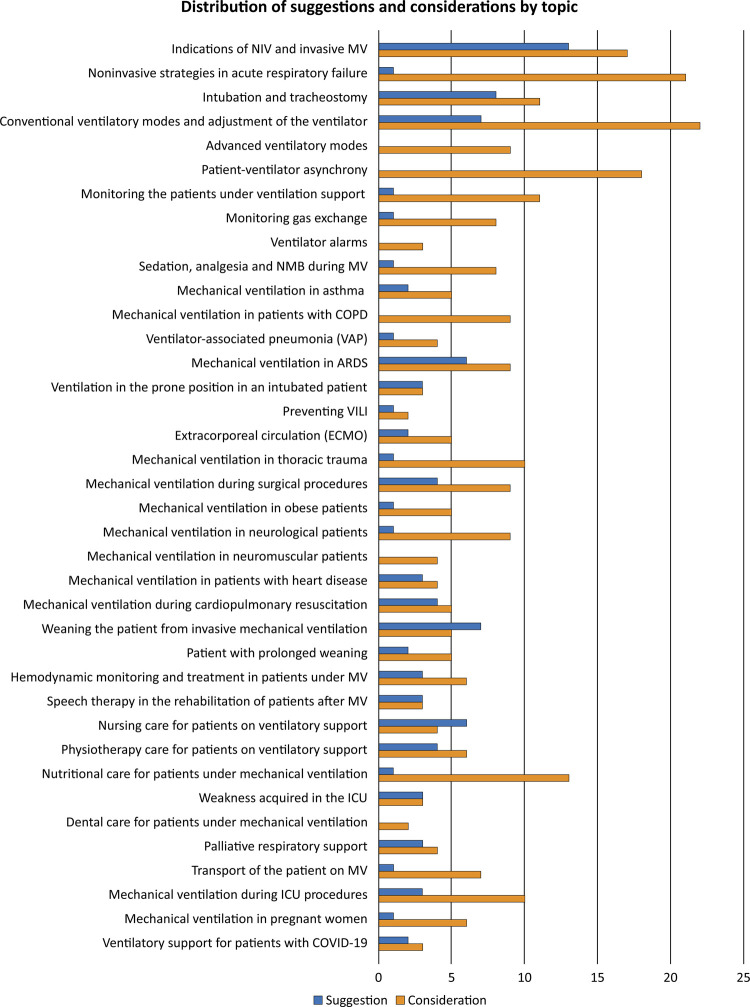
Number of suggestions (blue) and considerations (orange) by topic.

The experts made a total of 100 suggestions and 288 considerations in relation to the 38 themes (Figure 1). Consensus with a simple majority was reached during the plenary session for almost all suggestions/considerations, and electronic voting was required for four of the most controversial issues. Table 2 shows the most relevant suggestions and considerations for each topic and the four topics that required discussion. To access all the suggestions and considerations, please refer to the original document, which is freely available on the two societies’ websites (https://indd.adobe.com/view/017f739a-847f-4587-9bef-15b9c01756ba).

NIV - noninvasive ventilation; MV - mechanical ventilation; NMB - neuromuscular blocker; COPD - chronic obstructive pulmonary disease; ARDS - acute respiratory distress syndrome; VILI - ventilator-induced lung injury; ICU - intensive care unit.

### FINAL COMMENTS

The development of a practical bedside document and the updating of the previous Brazilian recommendations for mechanical ventilation led to a collaborative effort between AMIB and SBPT. The experts reviewed the latest evidence related to the care of patients undergoing MV, following the proposed methodology. This process generated suggestions and considerations, which were initially discussed and voted on in a plenary meeting and then reviewed by the organizing committee before being published. This document has been made publicly available and is being disseminated by both professional societies to provide guidance at the bedside across the country.

Clinical practice guidelines are considered valuable instruments for narrowing the gap between research findings and actual clinical practice.^(18-20)^ These tools enhance and standardize treatment, optimize patient care, and potentially reduce mortality rates and healthcare costs.^(21-23)^ but are still underutilized in clinical settings.^(24)^ Additionally, there is a need for locally developed clinical guidelines and treatment protocols in low- and middle-income countries (LMICs), as resource limitations may prevent the application of guidelines developed in high-resource settings.^(25)^ Simply translating guidelines and treatment protocols produced in high-resource settings is not enough, as the context in which they are applied is different.

The development of this bedside guide can help fill that gap. Providing guidance on a series of topics related to MV addresses an unmet need in an area with a high burden of disease.^(26,27)^A large observation study performed in 2013 in several Brazilian ICUs revealed that the mortality of patients under MV was higher than that in high income countries.^(26)^ During the COVID-19 pandemic, the strain imposed on an already overstressed healthcare system led to extremely high mortality in patients who required MV in Brazil.^(17,28-30)^Although worse outcomes have been reported across the globe, considerable variation has been reported, showing that some ICUs are more resilient and are able to adapt and respond to strain with less impact on patient outcomes.^(31)^ Among many components, a resilient ICU invests in the implementation of evidence-based practices and staff training. For example, the use of protective ventilatory strategies^(28)^ and timely use of noninvasive ventilation^(29)^ are associated with lower mortality, suggesting that the implementation of evidence-based strategies in MV has an impact on patient outcomes, especially in situations of strain.

We produced a comprehensive document addressing 38 topics related to ventilatory support. In almost three quarters of the cases, there were no randomized controlled trials to inform suggestions; therefore, the guidance to readers was less emphatic, with a consideration to use or not use a given intervention. Although the lack of robust evidence prevented us from providing more assertive suggestions on these topics, we believe that the considerations are valuable because evidence in the form of clinical trials is lacking for important topics such as choosing the mode of ventilation or how to adjust the initial settings of a ventilator, which are typically not addressed in clinical practice guidelines produced with methodologies such as GRADE. In the case of specific ventilatory strategies, such as prone positioning, recruitment maneuvers and the use of neuromuscular blockage, more than one randomized controlled trial was available, and a suggestion could be made. Notably, these topics are already covered by two recent clinical practice guidelines and recommendations in the same lines as our suggestions were made.^(9,10)^

If a lack of training, resulting in low confidence in managing patients under MV among clinicians,^(5,6)^ a lack of adoption of evidence-based strategies in MV^(1)^ and a lack of treatment protocols^(32,33)^ to facilitate the implementation of such strategies contribute to the greater burden of acute respiratory failure in LMICs, these gaps offer a significant opportunity for improvement in outcomes. The dissemination of evidence-based best practices in the form of accessible documents can offer guidance to clinicians at the bedside and inform treatment protocol development. Although the joint statement produced by AMIB and SBPT alone is not sufficient, emphasizing the urgent need for health care capacity building, specialization and training, investments in infrastructure, and other measures to improve healthcare systems and processes of care, it is an important first step.

Despite having been developed to meet the needs of the Brazilian critical care context, two major barriers remain. First, ensuring ample dissemination and consistent adoption.^(34)^ Healthcare professionals’ negative attitudes and beliefs, limited integration of guideline recommendations into organizational structures, time and resource constraints and organizational- and system-level changes are identified barriers.^(24)^ Second, inequalities in ICU resources across Brazil will impact the applicability of some of the suggestions and considerations made in the document. For example, we suggest that high-flow nasal cannulas can be used in a variety of scenarios because randomized controlled trials have shown that they are effective for avoiding intubation and reducing mortality in patients with respiratory failure, but many ICUs in Brazil do not have that technology readily available. The same can be said about the recommendation to use ECMO for refractory hypoxemia and expensive monitoring devices, such as end-tidal CO_2_ and indirect calorimetry. When preparing suggestions and considerations, we aimed to balance the availability of evidence in favor of such interventions and the Brazilian context, recognizing that although many ICUs in Brazil may not have access to interventions that include complex and/or expensive technology, when the evidence is strong in favor of the benefit they offer, it would not be appropriate to refrain from suggesting their use. On the contrary, we believe that the suggestion for use of evidence-based interventions stated in a document endorsed by two respected medical societies can help inform public health policy in Brazil, supporting the incorporation of technologies that have been shown to reduce mortality, such as noninvasive ventilation, high-flow nasal cannulas^(29)^ and ECMO.^(35)^

The present study has several limitations: the methodology adopted did not include performing systematic reviews and meta-analyses to make recommendations, as is the case with the GRADE methodology, because with so many topics, it would be impractical to adopt this strategy. In addition, we did not formally evaluate the quality of the studies, as the GRADE methodology typically does to formulate recommendations. The experts were instructed to use their own judgment when selecting references. As a result, it is possible that some of the studies used in the document were at high risk of bias. Therefore, no recommendations were made, and we used a different terminology, with suggestions and considerations. The decision to perform a focused review of each theme, instead of systematic reviews and meta-analyses with PICO questions, was made to allow the document to be as comprehensive as possible. The topics and their scopes were determined by the coordinators by informal consensus and were therefore subject to selection bias. In addition, some topics in the document were not examined in clinical trials; therefore, the considerations made about them were based on physiological studies or expert opinions. The document also has strengths: the topics were thoroughly evaluated by professionals recognized as experts in MV, and there was a plenary discussion of all the topics and voting, when necessary, highlighting the robustness of the suggestions and considerations formulated.

## CONCLUSION

Evidence-based and up-to-date guidance is essential to ensure that healthcare providers are informed by best practices for the management of patients undergoing mechanical ventilation. This joint statement aims to standardize care, reduce variability in clinical practice, improve patient outcomes, and support teaching in MV. Its implementation can lead to a decrease in complications associated with MV, optimization of the use of resources, and improvement in the quality of patient care.

**Table:** 

Authors of the Working Group of Mechanical Ventilation Practical Suggestions and Considerations	
Alexandre Biasi Cavalcanti	*Instituto de Pesquisa, HCor-Hospital do Coração* - São Paulo (SP), Brazil.
Ana Maria Casati Nogueira da Gama	*Hospital Universitário “Cassiano Antônio de Moraes”, Universidade Federal do Espírito Santo* - Vitória (ES), Brazil.
Angelo Roncalli Miranda Rocha	*Hospital Geral do Estado de Alagoas* - Maceió (AL), Brazil.
Antonio Gonçalves de Oliveira	*Complexo Hospitalar Unimed Recife* - Recife (PE), Brazil.
Ary Serpa-Neto	Australian and New Zealand Intensive Care Research Centre, Monash University - Melbourne, Victoria, Australia.
Augusto Manoel de Carvalho Farias	*Hospital Português* - Salvador (BA), Brazil.
Bianca Rodrigues Orlando	*Hospital Escola, Universidade Federal de Pelotas* - Pelotas (RS), Brazil.
Bruno da Costa Esteves	*Hospital Evangélico de Sorocaba* - Sorocaba (SP), Brazil.
Bruno Franco Mazza	*Hospital Israelita Albert Einstein* - São Paulo (SP), Brazil.
Camila de Freitas Martins Soares Silveira	*Hospital Israelita Albert Einstein Goiânia* - Goiânia (GO), Brazil.
Carlos Roberto Ribeiro de Carvalho	*Instituto do Coração, Hospital das Clínicas, Faculdade de Medicina, Universidade de São Paulo* - São Paulo (SP), Brazil.
Carlos Toufen Junior	*Instituto do Coração, Hospital das Clínicas, Faculdade de Medicina, Universidade de São Paulo* - São Paulo (SP), Brazil.
Carmen Silvia Valente Barbas	*Instituto do Coração, Hospital das Clínicas, Faculdade de Medicina, Universidade de São Paulo* - São Paulo (SP), Brazil.
Cassiano Teixeira	Universidade Federal de Ciências da Saúde de Porto Alegre - Porto Alegre (RS), Brazil.
Débora Dutra da Silveira	*Universidade Federal de São Paulo* - São Paulo (SP), Brazil.
Denise Machado Medeiros	*Instituto Nacional de Infectologia “Evandro Chagas”, Fundação “Oswaldo Cruz”* - Rio de Janeiro (RJ), Brazil.
Edino Parolo	*Hospital de Clínicas de Porto Alegre, Universidade Federal do Rio Grande do Sul* - Porto Alegre (RS), Brazil.
Eduardo Leite Vieira Costa	*Instituto do Coração, Hospital das Clínicas, Faculdade de Medicina, Universidade de São Paulo* - São Paulo (SP), Brazil.
Eliana Bernadete Caser	*Hospital Universitário “Cassiano Antônio de Moraes”, Universidade Federal do Espírito Santo* - Vitória (ES), Brazil.
Ellen Pierre de Oliveira	*Instituto do Coração, Hospital das Clínicas, Faculdade de Medicina, Universidade de São Paulo* - São Paulo (SP), Brazil.
Eric Grieger Banholzer	*Pontifícia Universidade Católica do Paraná* - Curitiba (PR), Brazil.
Erich Vidal Carvalho	*Hospital Universitário, Universidade Federal de Juiz de Fora* - Juiz de Fora (MG), Brazil.
Fabio Ferreira Amorim	*Escola Superior de Ciências da Saúde, Universidade do Distrito Federal* - Brasília (DF), Brazil.
Felipe Saddy	*Hospital Pró-Cardíaco* - Rio de Janeiro (RJ), Brazil.
Fernanda Alves Ferreira Gonçalves	*Hospital das Clínicas, Universidade Federal de Goiás* - Goiânia (GO), Brazil.
Filomena Regina Barbosa Gomes Galas	*Instituto do Coração, Hospital das Clínicas, Faculdade de Medicina, Universidade de São Paulo* - São Paulo (SP), Brazil.
Giovanna Carolina Gardini Zanatta	*Hospital Alvorada* - São Paulo (SP), Brazil.
Gisele Sampaio Silva	*Universidade Federal de São Paulo* - São Paulo (SP), Brazil.
Glauco Adrieno Westphal	*Centro Hospitalar Unimed* - Joinville (SC), Brazil.
Gustavo Faissol Janot de Matos	*Hospital Israelita Albert Einstein* - São Paulo (SP), Brazil.
João Cláudio Emmerich de Souza	*Hospital Federal dos Servidores do Estado* - Rio de Janeiro (RJ), Brazil.
João Manoel Silva Junior	*Faculdade de Medicina, Universidade de São Paulo* - São Paulo (SP), Brazil.
Jorge Luis dos Santos Valiatti	*Curso de Medicina, Centro Universitário Padre Albino* - Catanduva (SP), Brazil.
José Ribamar do Nascimento Junior	*Instituto de Gerenciamento em Fonoaudiologia e Deglutição* - São Paulo (SP), Brazil.
José Rodolfo Rocco	*Universidade Federal do Rio de Janeiro* - Rio de Janeiro (RJ), Brazil.
Ludhmila Abrahão Hajjar	*Faculdade de Medicina, Universidade de São Paulo* - São Paulo (SP), Brazil.
Luiz Alberto Forgiarini Junior	*Universidade Católica de Pelotas* - Pelotas (RS), Brazil.
Luiz Marcelo Sá Malbuisson	*Hospital das Clínicas, Faculdade de Medicina, Universidade de São Paulo* - São Paulo (SP), Brazil.
Marcelo Alcântara Holanda	*Universidade Federal do Ceará* - Fortaleza (CE), Brazil.
Marcelo Britto Passos Amato	*Instituto do Coração, Hospital das Clínicas, Faculdade de Medicina, Universidade de São Paulo* - São Paulo (SP), Brazil.
Marcelo Park	*Hospital das Clínicas, Faculdade de Medicina, Universidade de São* Paulo - São Paulo (SP), Brazil.
Marco Antônio da Rosa e Oliveira	*Departamento de Educação Continuada, Imed Group* - São Paulo (SP), Brazil.
Marco Antônio Soares Reis	*Hospital Madre Teresa* - Belo Horizonte (MG), Brazil.
Marcos Soares Tavares	*Hospital 9 de Julho* - São Paulo (SP), Brazil.
Mario Henrique Dutra de Souza	*Hospital Alvorada* - Rio de Janeiro (RJ), Brazil.
Marta Cristina Pauleti Damasceno	*Santa Casa de Misericórdia de São João da Boa Vista* - São Paulo (SP), Brazil.
Marta Maria da Silva Lira-Batista	*Hospital Universitário, Universidade Federal do Piauí* - Teresina (PI), Brazil.
Max Morais Pattacini	*Hospital da Bahia*, DASA - Salvador (BA), Brazil.
Murillo Santucci Cesar de Assunção	*Hospital Israelita Albert Einstein* - São Paulo (SP), Brazil.
Neymar Elias de Oliveira	*Fundação Faculdade Regional de Medicina de São José do Rio Preto* - São José do Rio Preto (SP), Brazil.
Oellen Stuani Franzosi	*Hospital de Clínicas de Porto Alegre, Universidade Federal do Rio Grande do Sul* - Porto Alegre (RS), Brazil.
Patricia Rieken Macedo Rocco	*Laboratório de Investigação Pulmonar, Instituto de Biofísica Carlos Chagas Filho, Universidade Federal do Rio de Janeiro* - Rio de Janeiro (RJ), Brazil.
Pedro Caruso	AC Camargo Center - São Paulo (SP), Brazil.
Pedro Leme Silva	*Laboratório de Investigação Pulmonar, Instituto de Biofísica Carlos Chagas Filho, Universidade Federal do Rio de Janeiro* - Rio de Janeiro (RJ), Brazil.
Pedro Vitale Mendes	*Hospital das Clínicas, Faculdade de Medicina, Universidade de São Paulo* - São Paulo (SP), Brazil.
Péricles Almeida Delfino Duarte	*Universidade Estadual de Ponta Grossa* - Ponta Grossa (PR), Brazil.
Renato Fábio Alberto Della Santa Neto	*Hospital das Clínicas, Universidade Federal de Pernambuco* - Recife (PE), Brazil.
Ricardo Goulart Rodrigues	*Hospital do Servidor Público Estadual “Francisco Morato de Oliveira”* - São Paulo (SP), Brazil.
Ricardo Luiz Cordioli	*Hospital Israelita Alberta Einstein* - São Paulo (SP), Brazil.
Roberta Fittipaldi Palazzo	*Instituto do Coração, Hospital das Clínicas, Faculdade de Medicina, Universidade de São Paulo* - São Paulo (SP), Brazil.
Rosane Goldwasser	*Hospital Universitário “Clementino Fraga Filho”, Universidade Federal do Rio de Janeiro* - Rio de Janeiro (RJ), Brazil.
Sabrina dos Santos Pinheiro	*Hospital Nossa Senhora Conceição, Grupo Hospitalar Conceição* - Porto Alegre (RS), Brazil.
Sandra Regina Justino	*Complexo do Hospital de Clínicas, Universidade Federal do Paraná* - Curitiba (PR), Brazil.
Sergio Nogueira Nemer	*Complexo Hospitalar de Niterói* - Rio de Janeiro (RJ), Brazil.
Vanessa Martins de Oliveira	*Hospital de Clínicas de Porto Alegre, Universidade Federal do Rio Grande do Sul* - Porto Alegre (RS), Brazil.
Vinicius Zacarias Maldaner da Silva	*Universidade de Brasília* - Brasília (DF), Brazil.
Wagner Luis Nedel	*Grupo Hospitalar Conceição* - Porto Alegre (RS), Brazil.
Wanessa Teixeira Bellissimo-Rodrigues	*Hospital das Clínicas, Faculdade de Medicina de Ribeirão Preto, Universidade de São Paulo* - Ribeirão Preto (SP), Brazil.
Wilson de Oliveira Filho	*Hospital Universitário “Getúlio Vargas”, Universidade Federal do Amazonas -* Manaus (AM), Brazil.
